# Noninvasive Ultrasound Molecular Imaging of the Effect of Statins on Endothelial Inflammatory Phenotype in Early Atherosclerosis

**DOI:** 10.1371/journal.pone.0058761

**Published:** 2013-03-15

**Authors:** Elham Khanicheh, Martina Mitterhuber, Lifen Xu, Stéphanie P. Haeuselmann, Gabriela M. Kuster, Beat A. Kaufmann

**Affiliations:** 1 Department of Biomedicine, University of Basel and University Hospital Basel, Basel, Switzerland; 2 Cardiology, University Hospital Basel, Basel, Switzerland; University Medical Center Utrecht, Netherlands

## Abstract

**Background/Objectives:**

Inflammatory changes on the endothelium are responsible for leukocyte recruitment to plaques in atherosclerosis. Noninvasive assessment of treatment-effects on endothelial inflammation may be of use for managing medical therapy and developing novel therapies. We hypothesized that molecular imaging of vascular cell adhesion molecule-1 (VCAM-1) with contrast enhanced ultrasound (CEU) could assess treatment effects on endothelial phenotype in early atherosclerosis.

**Methods:**

Mice with atherosclerosis produced by gene deletion of the LDL-receptor and Apobec-1-editing protein were studied. At 12 weeks of age, mice received 8 weeks of regular chow or atorvastatin-enriched chow (10 mg/kg/day). At 20 weeks, CEU molecular imaging for aortic endothelial VCAM-1 expression was performed with VCAM-1-targeted (MB_VCAM_) and control microbubbles (MB_Ctr_). Aortic wall thickness was assessed with high frequency ultrasound. Histology, immunohistology and Western blot were used to assess plaque burden and VCAM-1 expression.

**Results:**

Plaque burden was reduced on histology, and VCAM-1 was reduced on Western blot by atorvastatin, which corresponded to less endothelial expression of VCAM-1 on immunohistology. High frequency ultrasound did not detect differences in aortic wall thickness between groups. In contrast, CEU molecular imaging demonstrated selective signal enhancement for MB_VCAM_ in non-treated animals (MB_VCAM_ 2±0.3 vs MB_Ctr_ 0.7±0.2, p<0.01), but not in statin-treated animals (MB_VCAM_ 0.8±0.2 vs MB_Ctr_ 1.0±0.2, p = ns; p<0.01 for the effect of statin on MB_VCAM_ signal).

**Conclusions:**

Non-invasive CEU molecular imaging detects the effects of anti-inflammatory treatment on endothelial inflammation in early atherosclerosis. This easily accessible, low-cost technique may be useful in assessing treatment effects in preclinical research and in patients.

## Introduction

Large primary and secondary prevention trials have consistently shown a risk reduction for cardiovascular events when patients with established vascular disease, diabetes or hyperlipidemia are treated with statins [1]–[3]. In addition, a specific subset of patients with low LDL cholesterol levels and elevated high sensitive C-reactive protein also benefits from statin treatment [4]. However, statin treatment confers a risk reduction for cardiovascular events of 40% at best [5], thus leaving the majority of events to occur despite treatment. This residual risk may be attributable to incomplete reduction in inflammatory status despite reaching LDL-goals. Improved strategies for atherosclerosis treatment will likely include better risk assessment tools that allow for risk stratification and treatment during the early stages of atherosclerosis, and additional pharmacologic interventions that target inflammation. For pharmacologic interventions that are started at early timepoints during the pathogenesis of atherosclerosis, or that include novel, costly drug regimens with the goal of reducing vascular inflammation, the ability to non-invasively assess treatment effect on vascular inflammatory status will therefore be important.

Endothelial expression of the inflammatory cell adhesion molecule VCAM-1 plays an important role in the pathogenesis of atherosclerosis by regulating leukocyte recruitment to nascent atherosclerotic plaques in the arterial wall [6], and assessment of VCAM-1 expression may be a good target for testing the adequacy of anti-inflammatory treatment effects both for preclinical research in animal models, but also in humans. Ultrasound molecular imaging is a low-cost, easily accessible screening method that has been shown to be feasible for the assessment of VCAM-1 expression during early atherosclerosis [7], and thus may allow for assessment of the adequacy of anti-inflammatory treatment in early atherosclerosis.

In the present study, we therefore investigated whether *in vivo* ultrasound molecular imaging of the expression of VCAM-1 could be used to assess the effects of pharmacologic therapy on vascular endothelial phenotype in a mouse model of *early* atherosclerosis. To examine how molecular imaging of the endothelial phenotype compares to assessment of vessel wall morphology, high frequency ultrasound imaging of the aortic arch was also performed.

## Methods

### Mouse Model

All experiments were performed in accordance with Swiss Federal Legislation and were approved by the local Animal Care and Use Committee of the University Hospital of Basel and the ethics committee of the Veterinary Office of the Canton of Basel. Mice with a double knockout for the LDL receptor and the Apobec-1 editing enzyme on a C57Bl/6 background were used. These mice develop atherosclerosis in a predictable, time dependent fashion while on a normal chow diet [8]. At 12 weeks of age, the mice were put either on a chow diet containing 0.01% (wt/wt) atorvastatin, resulting in a dose of approximately 10 mg/kg/day (n = 19) or an identical chow diet without atorvastatin (n = 18). At 20–22 weeks of age, mice were anesthetized with inhaled isoflurane (1 to 2%), and a jugular intravenous catheter was placed for administration of microbubbles. The adequacy of anesthesia was regularly assessed with toe-pinch, and the dose of inhaled isoflurane adjusted if necessary. High frequency ultrasound and ultrasound molecular imaging for the expression of VCAM-1 were performed. Subsequently, blood from randomly selected animals (n = 7) from the statin group, n = 6 from the non-treated group) was collected into EDTA-coated tubes by puncture of the left ventricle and was centrifuged at 4°C for 20 min at 2800 rpm to obtain serum. Total cholesterol, combined low density lipoprotein (LDL) and very low density lipoprotein (VLDL) cholesterol as well as high density lipoprotein (HDL) cholesterol were measured using a commercial assay kit (EHDL-100, BioAssay-Systems, Hayward, CA). Animals were sacrificed by cervical dislocation under deep sedation. The aortas were harvested for histology, fluorescent immunohistochemistry, and VCAM-1 western blotting.

### Microbubble Preparation

Biotinylated, lipid-shelled decafluorobutane microbubbles were prepared by sonication of a gas saturated aqueous suspension of distearoylphosphatidylcholine (2 mg/ml; Avanti Polar Lipids, Alabaster AL), polyoxyethylene-40-stearate (1 mg/ml; Sigma), and 1,2-distearoyl-sn-glycero-phosphoethanolamine-N-[biotinyl(polyethylene glycol)-3400] (0.15 mg/ml, Creative PEG Works, Winston Salem, NC). Microbubbles targeted to VCAM-1 (MB_VCAM_) were prepared by conjugation of biotinylated rat anti-mouse VCAM-1 antibody (MK 2.7) to the microbubble surface using biotin-streptavidin-biotin linking as previously described [9]. Control microbubbles (MB_Ctr_) bearing a non-specific isotype control antibody (R3–34, BD Bioscience) were also prepared. Microbubble concentration and size were measured by electrozone sensing (Multisizer III, Beckman- Coulter). There was no size difference between MB_VCAM-1_ and MB_Ctr_ (3.2 µm ±0.1 vs. 3.1 µm ±0.1, p = ns).

### Contrast Enhanced Ultrasound Molecular Imaging

Ultrasound imaging (Sequoia Acuson C512; Siemens Medical Systems USA Inc., Mountain View, CA) was performed with a high-frequency linear-array probe (15L8) held in place by a railed gantry system. The ascending aorta of the mouse was imaged in a long axis plane from a right parasternal window, care was taken to include the sinus of valsalva and the take-off of the brachiocephalic artery in the image. Contrast enhanced ultrasound (CEU) was performed with power modulation and pulse inversion (Contrast Pulse Sequence) imaging at a centerline frequency of 7 MHz and a dynamic range of 50 dB. The gain settings were adjusted to levels just below visible noise speckle and held constant. MB_VCAM_ or MB_Ctr_ (1×10^6^ microbubbles per injection) were injected intravenously via a cannulated jugular vein in random order, while ultrasound imaging was paused. Eight minutes after microbubble injection, imaging was resumed at a mechanical index of 0.87. The first acquired image frame was used to derive the total amount of microbubbles present within the aorta. The microbubbles in the ultrasound beam were then destroyed with several (>10) image frames. Several image frames at a long pulsing interval (10 sec) were then acquired to measure signal attributable to freely circulating microbubbles. Data were log-linear converted, and frames representing freely circulating microbubbles were digitally subtracted from the first image to derive signal from attached microbubbles alone. Contrast intensity was measured from a region of interest encompassing the sinus of valsalva, the ascending aorta and the initial portion of the aortic arch, extending into the origin of the brachiocephalic artery. The selection of the region of interest was guided by fundamental frequency anatomic images of the ascending aorta acquired at 14 MHz at the end of each individual imaging sequence.

### High Frequency Ultrasound Assessment of Plaque Size and Cardiac Function

High frequency imaging of the aortic arch in long axis was performed with a Vevo 2100 (Visual Sonics Inc.) imaging system equipped with an MS550D transducer operating at 40 MHz at the shortest possible pulse length. This system has an axial resolution of 40 µm [10]. Plaque size was assessed by measuring the vessel wall thickness along the axial orientation of the ultrasound beam on real time images at the sinus of valsalva, the greater curvature, the lesser curvature, and at the take off of the brachiocephalic artery by an investigator blinded to animal treatment. M-Mode images of the left ventricle at the height of the papillary muscles were used to calculate fractional shortening, aortic peak flow velocity was measured on pulsed wave spectral doppler tracings from the aortic arch.

### Histology

All histological preparations were done on sections from the base of the aorta at the sinus of valsalva and from the ascending aorta at the take off of the brachiocephalic artery. Due to the limited amount of tissue available and different embedding techniques, it was not possible to perform every histological assessment in every animal. For assessment of plaque burden, Movat’s pentachrome staining was performed. Paraffin-embedded aortas were sectioned in short axis from the aortic valve to the distal portion of the ascending aorta at the take off of the brachiocephalic artery and stained using a commercially available Movat’s pentachrome staining kit (F-384, Rowley Biochemical Inc). Histological plaque burden was measured as the plaque area in relation to the vessel area defined by the internal elastic lamina using Image-J.

Fluorescent immunohistochemistry was performed to qualitatively assess the endothelial expression of VCAM-1. Frozen aortic sections were mounted on glass slides, fixed in −20°C Acetone, air-dried, blocked with 10% goat serum in TBS/FSGO and incubated overnight at 4°C with anti-VCAM-1 (CBL-1300, Millipore). Goat anti-rat Alexa-633 (A21094, Invitrogen) was added and incubated for 1 hour at room temperature. Sections were mounted with Prolong gold antifade mounting medium and imaged on a Zeiss LSM 710 confocal microscope.

Immunostaining for Mac-2 was performed on paraffin-embedded aortic sections after heat-induced antigen retrieval with 10 mM NaCitrate Buffer (pH = 6). Sections were blocked with 3% BSA in PBS and incubated overnight at 4°C with anti-Mac-2 (ACL8942AP, lucerna-chem). Goat anti-rat Alexa-633 (A21094, Invitrogen) was added and incubated for 1 hour at room temperature. Sections were mounted with Prolong gold antifade mounting medium containing DAPI (P36935, Invitrogen) and imaged on a Zeiss LSM 710 confocal microscope. For quantitative immunocytochemical comparisons of macrophage content, the number of positively stained pixels was counted on thresholded pictures and normalized to total number of pixels of the vessel area encompassed by the internal elastic lamina using Image-J. Thresholds were defined as the mean background intensity plus 40 times the standard deviation in each individual picture. For each mouse at least two sections on different slides were imaged and quantified for both the base and ascending aorta.

### Western Blotting

Western blotting for the expression of VCAM-1 was performed in 5 statin treated animals and 5 non-treated animals that had not undergone ultrasound imaging. The ascending portion of the aorta was homogenized in lysis buffer (Cell Signaling) containing 80 mmol/L Pefabloc SC plus (Roche). Protein concentration was measured using the Micro BCA (bicinchoninic acid) protein assay kit (Thermo Scientific). 10 µg of protein were resolved on SDS-PAGE and transferred to Polyvinylidene fluoride (PVDF) membranes (Amersham). Membranes were probed with monoclonal rat anti-mouse VCAM-1 (Clone # 112702, R&D Systems) and monoclonal anti-α-tubulin (Clone DM1A, Sigma) antibodies. Blots were subsequently incubated with horseradish peroxidase-conjugated secondary antibodies (Jackson Immuno Research) and band intensities were detected by enhanced chemiluminescence (Western Lightening Plus; Perkin Elmer) and quantitated using NIH ImageJ software (http://rsbweb.nih.gov/ij/).

### Statistical Analysis

Data were analyzed on GraphPad Prism (version 5.0d). Data are expressed as mean±SEM unless stated otherwise. Single comparisons between the two animal groups were performed with a Mann Whitney test. Friedman’s repeated measures ANOVA with Dunn’s post hoc test was used to compare differences in microbubble signals within and between animal groups. A p value <0.05 (2-sided) was considered statistically significant.

## Results

### Effect of Statin Treatment on Cholesterol Levels

Statin treatment did not significantly influence total cholesterol or HDL-cholesterol levels, despite a trend towards decreased levels for both (table 1). In contrast, statin treatment resulted in a modest, but statistically significant 20% reduction in VLDL+LDL cholesterol levels.

**Table 1:** Effect of statin treatment on serum lipid profile (mg/dl) in LDL−/−Apobec1−/− mice (n = 7 statin group, n = 6 non-treated group).

	Non-treated	Statin	P- value
**Total cholestrol**	290.5±11.5	261.5±19.4	ns
**LDL+VLDL**	233±6.7	189.1±18.2	0.03
**HDL**	53.8±3.8	48.2±3.9	ns

### Histologic Plaque Area

**Figure 1:**
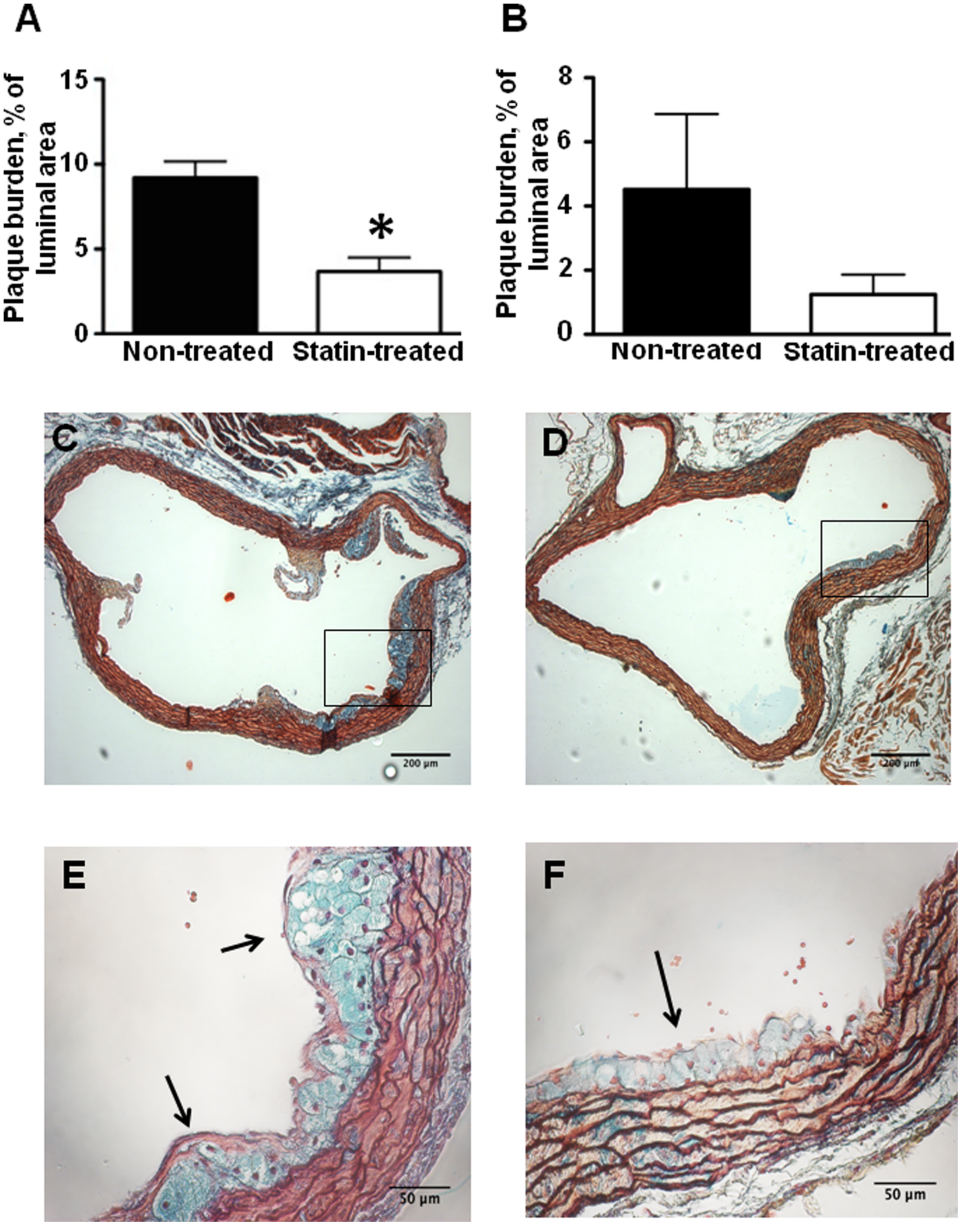
Effect of statin treatment on aortic atherosclerotic plaque burden. Percent of total luminal plaque area at the base of aorta (**A**) and in the ascending aorta (**B**) in non-treated animals versus statin treated animals (**A**, n = 11 non-treated animals, n = 7 statin treated animals; **B**, n = 7 non-treated and n = 7 statin treated animals), *p<0.05 vs non-treated animals. Examples of Movat’s pentachrom stains at the base of aorta in a non-treated mouse (**C, E**) and a statin treated mouse (**D, F**). The arrows denote a large plaque in a magnified view from a non-treated animal (**E**) and a small plaque in a magnified view from a statin treated animal (**F**).

Movat’s Pentachrome stains in non-treated animals showed macrophage-rich type III atherosclerotic lesions in the sinus of valsalva that occupied close to 8% of the vessel lumen area. Statin treatment resulted in a significant reduction in plaque burden in the sinuses of valsalva. In the ascending aorta, there was a similar, albeit statistically nonsignificant reduction in plaque area in animals that were receiving statin treatment (Figure 1).

### Expression of VCAM-1 and Vascular Inflammation

**Figure 2:**
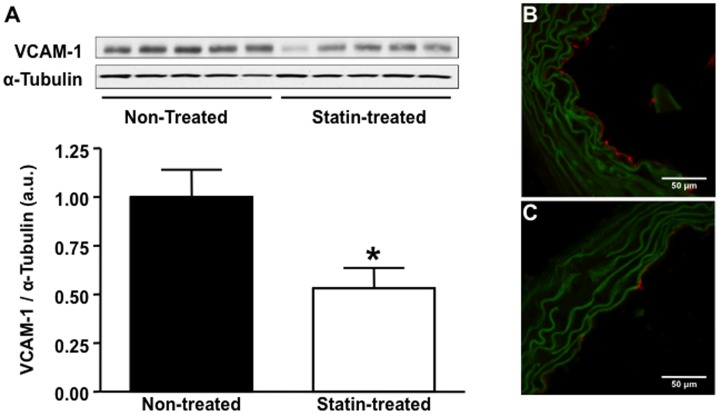
Effect of statin treatment on aortic expression of VCAM-1. (**A**) VCAM-1 protein expression in the ascending aorta assessed by Western blot in non-treated (lanes 1–5) versus statin treated (lanes 6–10) animals, n = 5 per group, *p<0.01 vs non-treated animals. Representative examples of fluorescent immunohistochemistry images of the base of the aorta demonstrating endothelial VCAM-1 expression (red fluorescence) in a non-treated animal (**B**) and a statin treated animal (**C**), the green fluorescence is autofluorescence delineating vessel anatomy.

**Figure 3:**
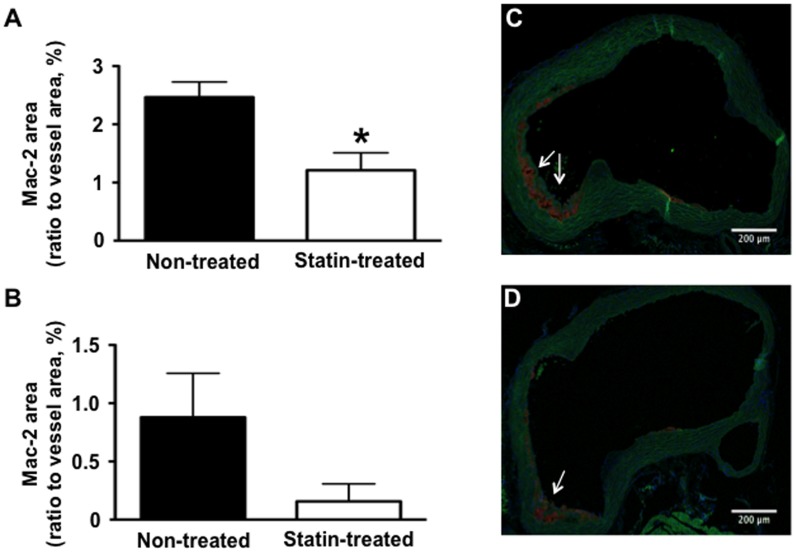
Effect of statin treatment on vascular inflammation. Fluorescent immunohistochemistry for the expression of macrophage Mac-2 at the base of the aorta (**A**) and in the ascending aorta (**B**) of non-treated versus statin treated mice (**A**, n = 12 non-treated animals, n = 9 statin treated animals; **B**, n = 8 non-treated and n = 8 statin treated animals), *p<0.05 versus non-treated animals. Examples of Mac-2 staining in a non-treated mouse (**C**) and a statin treated mouse (**D**), arrows denote stained Mac-2 positive macrophages.

Expression of VCAM-1 on Western blot was reduced by 47% (Figure 2A) in statin-treated animals. On immunohistochemistry, VCAM-1 staining was intense on endothelial cells both in plaque areas, but also on endothelium overlying apparently normal wall areas, and on macrophages inside plaques in non-treated animals. In contrast, endothelial staining was less pronounced on the endothelium in statin treated animals (Figure 2B and Figure 2C). Statin treatment resulted not just in a decrease in plaque area, but also in reduced macrophage content of plaques as assessed by Mac-2 staining (Figure 3A and 3B).

### High Frequency Ultrasound Assessment of Plaque Size and Cardiac Function

**Figure 4:**
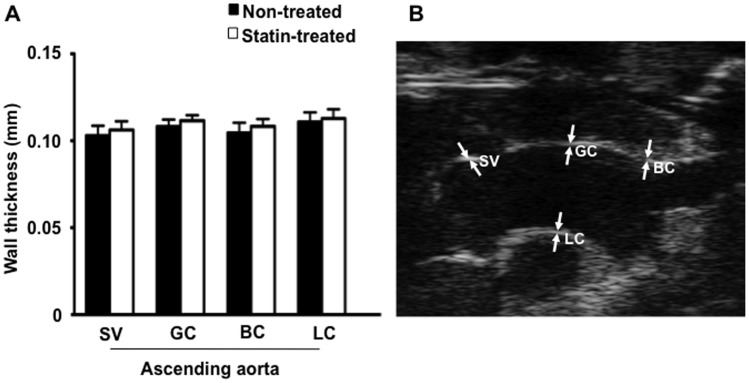
High frequency ultrasound imaging (40 MHZ) of the ascending aorta. (**A**) Wall thickness of the ascending aorta measured at the sinus of valsalva (SV), the greater curvature (GC), the lesser curvature (LC) and at the origin of brachoicephalic artery (BC) in non-treated and statin treated animals (n = 10 per group). (**B**) Example of high frequency ultrasound imaging of the ascending aorta illustrating the measurements obtained.

The aortic wall thickness was measured at atherosclerosis-prone sites throughout the ascending aorta. Despite histological differences in vessel wall thickness (59±22 µm) between statin treated and non-treated animals in plaque areas in the sinuses of valsalva that were above the theoretical axial resolution of the ultrasound system, blinded assessment of vessel wall thicknessess did not differ between the treatment groups at any of the measured points (Figure 4).

Left ventricular fractional shortening, aortic diameter, and aortic peak flow velocity did not systematically differ between statin treated and non-treated animals, indicating that shear rate conditions that oppose selective microbubble attachment in the ascending aorta did not differ between animal groups (Table 2).

**Table 2:** Echocardiographic data (mean ±1 SD, n = 13 in each group).

	Non-treated	Statin	P- value
**Fractional shortening (%)**	35.0±2.7	36.8±3.1	ns
**Aortic Internal Diameter (mm)**	1.3±0.1	1.3±0.1	ns
**Aortic Peak Systolic Velocity (m/s)**	0.63±0.15	0.61±0.10	ns

### Molecular Imaging of VCAM-1

**Figure 5:**
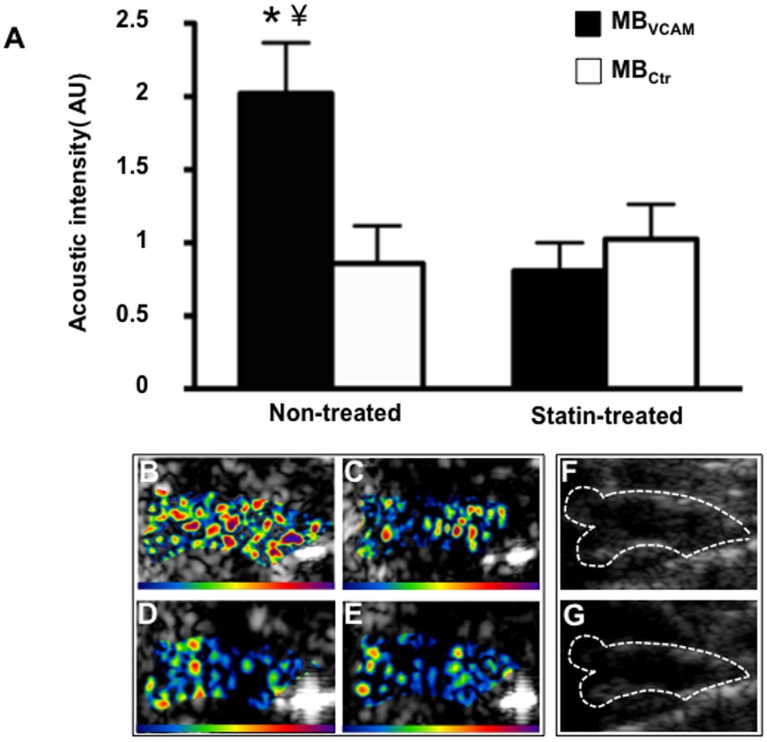
Molecular imaging of the ascending aorta. (**A**) Mean ± SEM background-subtracted signal intensity for microbubbles targeted to VCAM-1 (MB_VCAM_) and control microbubbles (MB_Ctr_) in non-treated (n = 10) and statin treated animals (n = 12). *p<0.01 vs MB_ctr_ in non-treated animals, ¥ p<0.01 vs MB_VCAM_ in statin treated animals. Examples of color coded CEU images from a non-treated animal after injection of MB_VCAM_ (**B**), and of MB_Ctr_ (**C**). Images from a statin treated animal after injection of MB_VCAM_ (**D**), and of MB_Ctr_ (**E**). The color scale for the CEU images is shown at the bottom of each frame. (**F**) and (**G**) illustrate the outline of the ascending aorta on B-mode ultrasound images which was used as a region of interest for acoustic intensity measurements.

CEU molecular imaging showed selective signal enhancement for microbubbles targeted to VCAM-1 in non-treated animals, with a 120% signal increase relative to signal from control microbubbles in the same animal group. In contrast, signal for microbubbles targeted to VCAM-1 was not increased over control microbubble signal in animals on statin treatment (Figure 5).

**Figure 6:**
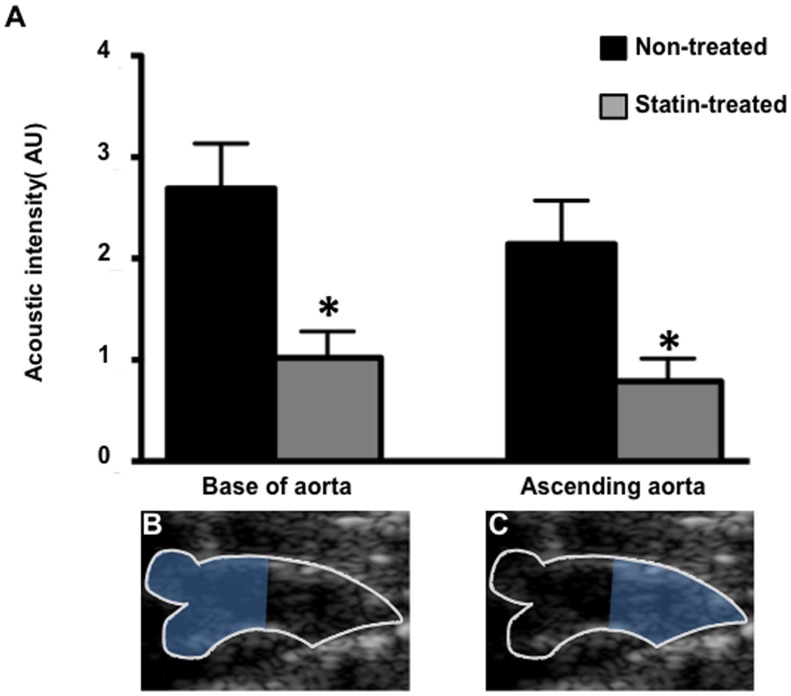
CEU molecular imaging of the regional endothelial expression of VCAM-1. (**A**) Mean ± SEM background-subtracted signal intensity for microbubbles targeted to VCAM-1 at the base of the aorta and in the distal ascending aorta in non-treated (n = 9) and statin treated animals (n = 10) *p<0.05 vs non-treated animals. Two dimensional ultrasound imaging illustrating outline of *(* **B**) base of the aorta and (**C**) ascending aorta.

Given the differences in plaque burden between the sinus of valsalva and the distal ascending aorta on histology, in an exploratory analysis, we also assessed the regional effect of statin treatment on CEU molecular imaging signal for microbubbles targeted to VCAM-1. In non-treated animals, signal for VCAM-1 targeted microbubbles was slightly, but not significantly lower in the distal ascending aorta compared to the base of the aorta. In both regions of interest, statin treatment resulted in a similar reduction in signal with significantly lower signals than in animals in non-treated animals (Figure 6).

## Discussion

Current treatment of atherosclerosis is to a large extent directed at patients with established clinical disease. However, it can be anticipated that in the future existing and emerging therapeutic agents will be employed at earlier stages during the disease process with the goal of quelling the molecular pathways that lead to the buildup of plaques, including endothelial recruitment of inflammatory cells. Such strategies will also create the need for a method for early, noninvasive detection of treatment effects. The results from our study indicate that ultrasound molecular imaging can detect the impact of therapies aimed at reducing endothelial inflammation during early disease stages in a murine model of atherosclerosis when high frequency morphologic plaque imaging is unable to detect a treatment effect.

The mouse model of atherosclerosis that we studied is characterized by a progressive development of atherosclerotic plaques with the first minimal lesions detected around 10 weeks of age, when treatment was started in our study. At 20 weeks of age, the mouse model shows lesions that cover about 5% of the total aortic surface, but 25–30% of the proximal aorta [7] in non-treated animals. The histologic changes found in early atherosclerosis such as in our mouse model are characterized by the accumulation of macrophages in the intima of the arterial wall. Recruitment of monocytes from the blood stream is tightly regulated by cell adhesion molecules expressed on the vascular endothelium, and VCAM-1 is essential for the development of early atherosclerotic lesions [11]. VCAM-1 is expressed on endothelial cells during the earliest stages of atherosclerosis [12], and has been shown to be an ideal target for ultrasound molecular imaging of vascular inflammation in both early and established atherosclerosis [7], [13]. Statin treatment reduces vascular inflammation and the expression of VCAM-1 on the vascular endothelium. This effect is mediated through a reduction in LDL-cholesterol [14], but also independent of the effects on serum cholesterol levels [15]. VCAM-1 is not stored in endothelial cells, and its expression on vascular endothelial cells depends on *de novo* synthesis of the protein. Regulation of VCAM-1 gene transcription involves the activation of NF-kB as a response to inflammatory stimuli. Statin treatment has been shown to reduce the activation NF-kB in endothelial cells [16] and thus attenuate the transcription of VCAM-1 mRNA.

The effect of statins in different mouse models of atherosclerosis has been variable [17]. In the mouse model that we used in our study, statin treatment resulted in a modest reduction in serum cholesterol levels. However, we found a more pronounced slowing of the progression of atherosclerotic plaque development, a reduction in VCAM-1 expression and reduced vascular inflammation, consistent with the aforementioned pleiotropic effects of statins on inflammatory pathways.

It has previously been shown that molecular imaging with targeted MRI and SPECT probes can be used for assessing the effect of statins in animal models of advanced atherosclerosis [18], [19]. Our study is the first to show that ultrasound molecular imaging is capable of specifically detecting the effects of pharmacologic treatment on endothelial inflammatory phenotype during the very early stages of the pathogenesis of atherosclerosis. It should be noted that, as opposed to molecular imaging performed with MRI and SPECT that relies on tracer retention not only on the endothelium but also within plaques, ultrasound molecular imaging specifically detects tracer retention on the vascular endothelial surface. Given the important role that endothelial expression of cell adhesion molecules has during the initial pathogenesis of atherosclerosis, an imaging approach that is capable of assessing endothelial phenotypic changes with high sensitivity is well suited not only for early detection [7] but also for assessing treatment effects during the initial stages of atherosclerosis. Similar to ultrasound carotid intima media thickening that has been used for risk assessment in clinical studies, we also performed high resolution ultrasound imaging with the intent to measure the effect of statin therapy on aortic wall thickness. Despite differences in plaque thickness on histology that were in the range of the theoretical axial resolution (40 µm) of the ultrasound system, no differences in wall thickness were detected on ultrasound imaging. In contrast, ultrasound molecular imaging was able to detect the effect of statin treatment on endothelial inflammatory phenotype. This was true for both the proximal ascending aorta, but also for the distal ascending aorta, which on histology showed a lower plaque area. The lower signal for microbubbles targeted to VCAM-1 in statin treated animals was associated not only with a reduction in VCAM-1 expression, but also with a reduced vascular inflammation as evidenced by macrophage Mac-2 staining.

Several limitations of our study should be mentioned. We used high-dose statin treatment in our animal model, and did not evaluate whether ultrasound molecular imaging would be capable of detecting graded responses to several different doses of statin treatment. As we did not perform examinations at multiple timepoints, we cannot comment on whether changes in vascular inflammatory phenotype precede changes in histology. Further it has been shown that microbubble targeting efficiency not only depends on the expression of target molecules, but also on endothelial glycocalyx thickness [20], which has been shown to be perturbed in atherosclerotic vessels, and can be partially restored by statin therapy [21]. Therefore, the effect on VCAM-1 signal in statin treated animals might also partially be due to a reduced targeting efficiency of MB_VCAM_ on the aortic endothelial surface. Due to the small size of the murine model of atherosclerosis in our study, the expression of VCAM-1 was imaged in the aorta. With regards to translation into humans, the aorta will not be a suitable vessel for imaging, nor will it be likely that noninvasive ultrasound molecular imaging of the coronary arteries will be feasible in the near future. However, the carotid artery is an accepted surrogate vessel for the assessment of cardiovascular risk and will be easily accessible for ultrasound molecular imaging in humans. Given the differences in plaque burden in the sinuses of valsalva versus the ascending aorta, we performed a regional analysis of VCAM-1 targetd signal. This analysis was not compared to regional assessment of VCAM-1 expression, in particular Western blot, and is to be viewed as an exploratory analysis. Last, from our study we cannot draw conclusions on whether the assessment of treatment effects early during the pathogenesis of atherosclerosis would be of value in improving disease management.

In conclusion, we show that ultrasound molecular imaging can detect the effects of statin therapy on early inflammatory processes in atherosclerosis at a timepoint when high-resolution imaging is unable to ascertain differences in plaque development. Such a low-cost, easily accesible imaging strategy could in the future be useful in the future for assessing the effect of drug treatment in individual patients, but also for screening of the effect of novel drug classes other than statins in the preclinical field.

## Acknowledgments

The authors would like to thank Dr. Jonathan Lindner for his scientific input and critical review of this manuscript.
